# The Association between Abstinence Period and Semen Parameters in Humans: Results in Normal Samples and Different Sperm Pathology

**DOI:** 10.3390/life14020188

**Published:** 2024-01-27

**Authors:** Min Xie, Silvan Hämmerli, Brigitte Leeners

**Affiliations:** 1 Department of Reproductive Endocrinology, University Hospital Zurich, 8091 Zurich, Switzerland; min.xie@usz.ch (M.X.); silvan.haemmerli@spitalzofingen.ch (S.H.); 2 Faculty of Medicine, University of Zurich, 8091 Zurich, Switzerland

**Keywords:** abstinence period, sperm quality, sperm concentration, sperm motility, sperm morphology, oligozoospermia, asthenozoospermia, teratozoospermia, oligoasthenoteratozoospermia

## Abstract

Background: The impact of sexual abstinence on sperm quality, particularly in pathological cases, is a subject of debate. We investigated the link between abstinence duration and semen quality in both normal and pathological samples. Methods: We analyzed semen samples from 4423 men undergoing fertility evaluation, comprising 1256 samples from healthy individuals and 3167 from those with conditions such as oligozoospermia, asthenozoospermia, teratozoospermia, or a combination of these factors, namely oligoasthenoteratozoospermia (OAT). Parameters including sperm concentration, the percentage of progressively motile spermatozoa, total motile sperm count, and the percentage of spermatozoa with normal morphology were assessed at various abstinence durations (each day, 0–2, 3–7, and >7 days). Results: Extended abstinence correlated with higher sperm concentration overall (*p* < 0.001), except in oligozoospermia. Longer abstinence reduced progressive motility in normal (*p* < 0.001) and teratozoospermic samples (*p* < 0.001). Shorter abstinence was linked to higher morphologically normal sperm in normal samples (*p* = 0.03), while longer abstinence did so in oligoasthenoteratozoospermic samples (*p* = 0.013). Conclusion: The findings suggest that a prolonged abstinence time is linked to higher sperm concentration, while optimal sperm motility is observed after shorter abstinence periods. However, results regarding morphology remain inconclusive. Recommendations on abstinence duration should be tailored based on the specific parameter requiring the most significant improvement.

## 1. Introduction

Semen analysis is a regular component of male fertility screening because male factors account for between 30% and 50% of cases of infertility [1,2]. Sperm quality affects not only naturally occurring conception but also the results of intrauterine insemination (IUI) and IVF (in vitro fertilization)/ICSI (intracytoplasmic sperm injection). Sperm concentration, the proportion of motile spermatozoa, and the proportion of morphologically normal spermatozoa are of special significance [3,4,5]. 

When the total motile sperm count (TMSC) is between 5 and 10 million (m), the success rates of IUI are much higher than when the TMSC is less than 5 million. Research on semen samples examined before IUI shows that a brief period of abstinence is linked to higher TMSC [6,7] as well as improved sperm motility, concentration, and the percentage of sperm with typical morphology [8]. The more subnormal semen characteristics in the first study, the greater the chance of a significant improvement in TMSC and total normal morphology count (TNMC) in a subsequent examination [6]. Conversely, most men exhibiting normal or minimally subnormal semen characteristics displayed declining values in their second ejaculate. There is, however, a dearth of information about the relationship between abstinence duration and various forms of semen disease. Sperm motility in IVF is positively correlated with rates of fertilization and pregnancy [3]. The significance of morphology, however, has been the subject of debate. While some studies have found a significant role for morphology in the likelihood of pregnancy, others have found very little relevance for morphology [9,10,11]. 

The World Health Organization (WHO) currently recommends an abstinence period of 2–7 days before semen analysis or prior to providing sperm for fertility treatments [12]. But the handbook does not include any background information on why this particular time window was chosen. In normozoospermic men, the duration of the abstinence period has been reported to affect sperm concentration, motility, and morphology. The optimal duration of abstinence, particularly for subfertile men, is a matter of debate. For example, the abstinence period may have distinct effects on semen parameters in men who are fertile and those who are not [13,14]. While some research indicates that abstinence periods of less than two days improve sperm concentration and motility in infertile men [8,15], other authors discovered that more than four days of abstinence led to higher motility [16,17]. 

Sperm count and TMSC increased 1.75 times after 5 days of abstinence compared with 1 day in a longitudinal research on six normozoospermic men [18], demonstrating the significant shift in sperm parameter values after varying abstinence intervals. On the other hand, following 1 day of abstinence, TMSC and TNMC quadrupled in most men with two or three subnormal sperm parameter values as opposed to 2–4 days [6]. 

Variations in abstinence time would represent a reasonably convenient and cost-effective way to improve semen parameters and associated chances for naturally conceived and medically induced pregnancy in men with abnormal semen parameters, even though such changes might not be clinically significant in normozoospermia. There are currently relatively few options to improve semen quality, other than treating infection and potentially varicosis [19]. This makes any solution that is accessible all the more crucial. Unfortunately, there is a current paucity of information regarding the significance of abstinence time for particular pathologies, such as oligo-, astheno-, terato- or oligoasthenozoospermia, where improving sperm quality is crucial to increasing chances of pregnancy. Lastly, if there is no correlation between the period of abstinence and the quality of sperm, the existing recommendation to abstain from sexual activity should be abandoned, which would lessen the psychological load on individuals dealing with infertility.

Hence, our study aimed to achieve two primary objectives: (i) assess potential variations in sperm quality following different durations of abstinence, and (ii) explore the relationship between the abstinence period and alterations in sperm parameters, specifically comparing men with normal semen samples to those with pathological parameters.

## 2. Materials and Methods

### 2.1. Study Design

This retrospective cohort study analyzed data derived from semen analyses conducted on men undergoing fertility evaluation at the andrology laboratory of the Department of Reproductive Endocrinology, University Hospital Zurich, Zurich, Switzerland. 

### 2.2. Patients

This study encompasses data from 4423 semen samples obtained from 4423 men who underwent their initial semen analysis between October 2003 and July 2015. Exclusions comprised men undergoing sperm cryopreservation before chemo- and/or radiation therapy for cancer. To eliminate potential confounding effects of antibiotics on semen quality, individuals who had taken antibiotics within the three months preceding the study period were also excluded. For multiple regression analysis, participants with any missing criteria in semen analysis (concentration, motility, and morphology) were excluded, along with those recording 0% for spermatozoa with normal forms (n = 458) or spermatozoa with normal motility (n = 19). Informed consent was obtained from all participants for inclusion in this study.

### 2.3. Semen Analysis

During the study period, 2 different editions of the WHO laboratory manual for the examination and processing of human semen were valid: the fourth edition and the fifth edition [12]. For the present study, we based the diagnosis of all semen analyses on criteria in the fifth edition. 

Semen samples were collected by masturbation directly at the andrology laboratory of the Department of Reproductive Endocrinology. Patients were advised by telephone and/or by a written information sheet to have no ejaculation for at least 2 days and for a maximum of 7 days prior to sample collection. When providing the sample, they received a questionnaire on confounders of semen analysis, which included a question on the number of days of abstinence prior to collecting the sample. Samples were analyzed within one hour after ejaculation. After liquefaction, semen volume was measured using a graduated serological pipet or a conical tube. Sperm concentration and motility were determined by counting the spermatozoa using a validated Makler chamber. All Makler chambers used in the andrology laboratory were validated using the Accu bead+ kit (Hamilton Thorne, Parallabs, UK). Motility was graded into 4 different categories: progressive motility (A: fast and B: slow progressive motility), non-progressive motility (C), and immotile spermatozoa (D). Vitality was assessed using the eosin–nigrosin test. Leukocytes were identified by checking for peroxidase-active cells in the semen samples. For the morphology assessment, a smear from each sample was air-dried and then fixed and stained, using the Papanicolaou staining method. Sperm morphology and immature germ cells were evaluated by visually inspecting at least 200 spermatozoa per sample using bright-field microscopy. Tygerberg strict criteria [20] were applied. The same team of trained laboratory technicians performed all assessments during the study period. Although cut-off values for normal parameters vary between the fourth and fifth editions of the WHO manual, analysis followed the criteria in the fifth edition throughout the study period. The laboratory participates regularly in an external quality control, QuaDeGA (quality control program of the German Society of Andrology). The semen parameters evaluated in the present study were (1) semen volume in milliliters, (2) sperm concentration in m/mL, (3) sperm motility, specifically the percentage of progressive motility, and the percentage of total motility (combination of progressive and non-progressive motility), and (4) total motile sperm count (calculated by multiplying the semen volume with the sperm concentration and the percentage of total motile spermatozoa for each sample). Additionally, (5) vitality, (6) percentage of spermatozoa with normal forms, (7) percentage of immature germ cells, and (8) the presence of leukocytes in m/mL were evaluated.

### 2.4. Definitions

Semen samples were categorized based on the lower reference values outlined in the fifth edition of the WHO laboratory manual. Those exceeding reference limits for sperm concentration, percentage of progressively motile spermatozoa, and percentage of normal forms were designated as “normal samples”. In cases where more than one semen parameter fell outside the normal range, the sample was included in the comparison for each respective “pathological” category against the normal samples.

### 2.5. Statistical Analysis

We evaluated abstinence time in days as a continuous variable and, to provide a basis for clearer clinical recommendations, also in different time categories, i.e., 0–2 days, 3–7 days, and >7 days.

After confirmation of adequate distribution of the sperm parameters, a Kruskal–Wallis test was conducted to determine if there were differences in the median semen parameter values among the samples with different abstinence times for different types of sperm pathology, i.e., normal, oligozoospermic, asthenozoospermic, teratozoospermic, and OAT samples. If median values were statistically significantly different among the abstinence interval groups, pairwise comparisons were performed using Dunn’s procedure with a Bonferroni correction for multiple comparisons. *p*-values < 0.05 were regarded as statistically significant. Differences in the number of immature germ cells between the different types of sperm pathology were first evaluated independently from the abstinence period with a Mann–Whitney U test and then investigated regarding the abstinence period. A multiple regression analysis was performed to evaluate associations between total abstinence time (days) and sperm concentration, total motile spermatozoa, and spermatozoa with normal morphology based on the different types of semen pathology. All statistical testing was completed with SPSS Statistics, Version 24.0 (IBM Corp. released 2016. IBM SPSS Statistics for Windows, Version 24.0., Armonk, NY, USA: IBM Corp).

## 3. Results

The mean age of the patients was 37.7 years (range 21–79 years) at the time of providing the sample. Descriptive statistics of the sperm parameters in normal and pathological semen analyses are presented in Table 1. 

Table 2 presents the regression analysis evaluating the association between the number of days of abstinence prior to semen collection and concentration, motility, and morphology in the total study group and in men receiving positive diagnoses of semen pathology. 

Table 3 shows the median values of the sperm parameters in each category of abstinence time for the normal samples.

**Figure 1:**
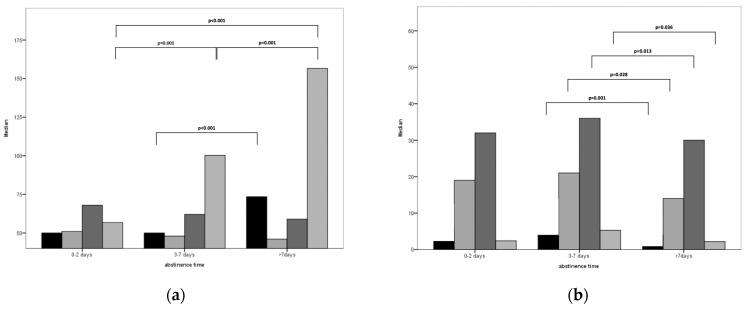

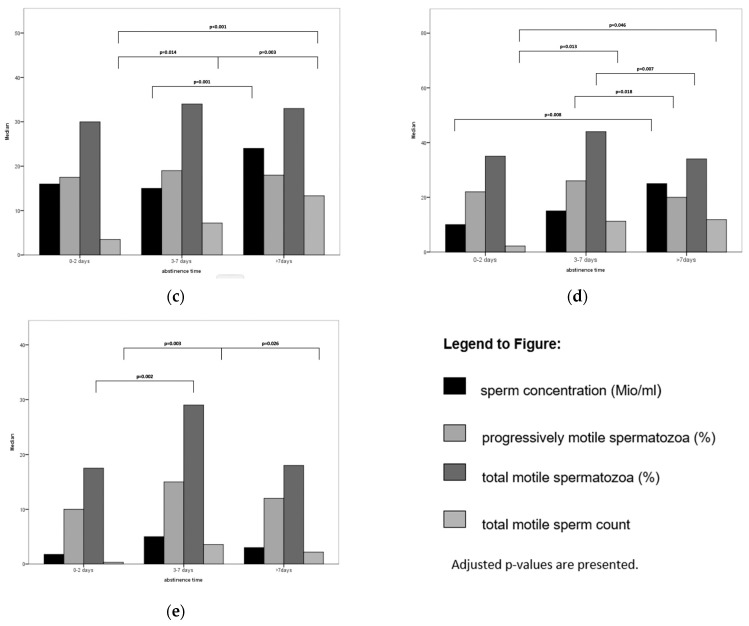
Median sperm parameters in relation to abstinence periods in different sperm pathology. (**a**) Normal samples; (**b**) oligozoospermic samples (73 men 0–2 days, 1594 men 3–7 days, 102 men > 7 days); (**c**) asthenozoospermic samples (26 men 0–2 days, 917 men 3–7 days, 55 men > 7 days); (**d**) teratozoospermic samples (25 men 0–2 days, 1155 men 3–7 days, 76 men > 7 days); (**e**) oligoasthenoteratozoospermic samples (10 men 0–2 days, 376 men 3–7 days, 19 men > 7 days).

Median values for oligo-, astheno-, teratozoospermic, and OAT samples in different categories of abstinence times are summarized in Figure 1. 

In normal semen samples, an extended abstinence period was associated with a higher sperm concentration, yet it led to a significant reduction in both progressive motility and normal morphology. When comparing results following 0–2, 3–7, and >7 days, the link between abstinence duration and sperm concentration was affirmed, with peak values observed for total motile sperm count (TMSC) after >7 days of abstinence. However, no discernible effect was observed for progressive motility and morphology in relation to the duration of abstinence. In cases of oligozoospermia, abstinence time did not show any association with sperm concentration, motility, or morphology. However, when evaluating different abstinence time categories, an interval exceeding 7 days exhibited a negative correlation with sperm concentration, total motile sperm count (TMSC), progressive motility, and overall motility. For asthenozoospermic samples, a longer abstinence time correlated with higher sperm concentration, but no significant differences were observed in motility or morphology. Subsequent analysis of distinct abstinence time categories confirmed an association between over 7 days of abstinence and elevated sperm concentration and TMSC, while no association was found with motility. Morphological parameters were optimal in semen samples collected after 0–2 days of abstinence. For teratozoospermia, associations with abstinence time were observed in concentration and motility. The highest concentration occurred after an abstinence period exceeding 7 days. TMSC was elevated after 3–7 days and beyond, with the peak motility observed after 3–7 days of abstinence. No association with morphology was identified in either analysis. For teratozoospermia, associations with abstinence time were observed in concentration and motility. The highest concentration occurred after an abstinence period exceeding 7 days. TMSC was elevated after 3–7 days and beyond, with the peak motility observed after 3–7 days of abstinence. No association with morphology was identified in either analysis. In the samples, a longer abstinence period correlated with higher sperm concentration and improved morphology when abstinence time was considered as a continuous variable. However, when comparing results in the three abstinence time categories, these findings were not confirmed. TMSC was significantly higher after 3–7 days and >7 days of abstinence. The median percentage of total motile spermatozoa was notably higher after 3–7 days compared to 0–2 days, while progressive motility showed no differences among the three abstinence time categories. 

## 4. Discussion

In summary, abstinence time is notably linked to sperm concentration, especially in normal semen samples. Motility and morphology exhibit diverse associations in both normal and pathological cases. Analyzing abstinence days as a continuous variable and comparing results within the 0–2, 3–7, and >7 days categories yielded similar outcomes in most but not all analyses.

### 4.1. Concentration, Volume, and TMSC

Our study aligns with a cross-sectional analysis of semen samples from normo- and oligozoospermic men, demonstrating comparable results for sperm concentration, volume, and total motile sperm count (TMSC) across various abstinence times (0–14 days) [15]. For normozoospermic samples in our study, both sperm concentration and TMSC peaked after 7 days of abstinence, surpassing the upper limit of the 2–7 days recommended by the WHO [12]. Previous evaluations of six consecutive sperm samples in normozoospermic men also revealed the highest sperm concentrations after >7 days of abstinence [18], a trend supported by smaller longitudinal studies [21,22].

The reduced sperm concentrations observed with shorter abstinence times in normozoospermic samples may be attributed to the depletion of stored spermatozoa in the cauda epididymis when ejaculation occurs more frequently [18]. Longer transport times through the epididymis, particularly in men with oligozoospermia where it was three times longer than in men with normozoospermia, are likely to impact sperm concentration [23]. In oligozoospermic samples, Levitas [15] reported a higher sperm concentration after 3–5 days and peak TMSC after 4 days of abstinence. These findings align with our results from assessing abstinence time categories but not with our continuous variable analysis of days of abstinence.

Beyond existing research findings, our study indicates that in astheno-, teratozoospermic, and OAT samples, semen concentration increases after longer abstinence periods. Enhancing sperm quality is crucial for fertility in cases of semen pathology, and an increase in concentration may help compensate for deficiencies in motility and morphology by augmenting the overall sperm count. However, contemporary research increasingly emphasizes sperm quality, which may not necessarily improve with higher concentration [24,25].

### 4.2. Motility

When assessing days of abstinence as a continuous variable, we observed that longer abstinence times were linked to reduced motility in both normal and teratozoospermic samples, consistent with previous research findings [15,26]. The oligo- and teratozoospermic samples exhibited the highest total motility values after 0–2 and 3–7 days of abstinence. However, asthenozoospermic and OAT samples, where an enhancement of motility would be essential for improving fertility, did not display significant differences concerning abstinence time categories. This contradicts the findings of Ayad et al. [25], who reported an increase in motility in normozoospermic samples after a short abstinence period and suggested that a shorter abstinence period might also enhance the motility of pathological sperm samples.

A plausible explanation for the favorable effects of shorter abstinence times could be related to the transit time of spermatozoa through the epididymis. More frequent ejaculation may result in shorter storage times of spermatozoa in the epididymis. During their residence in the epididymis, spermatozoa are exposed to reactive oxygen species (ROS) generated by the spermatozoa themselves and, to a lesser extent, by leukocytes. ROS can trigger lipid peroxidation of unsaturated fatty acids in the plasma membrane of spermatozoa [27,28]. Several studies have reported unusually high levels of ROS in infertile men [27,29]. It is proposed that excessively high ROS levels cannot be adequately counteracted by antioxidants in seminal plasma, such as catalase or superoxide dismutase, leading to oxidative stress, which negatively affects sperm motility [28]. Interestingly, the seminal total antioxidant capacity was notably higher after 1 day compared to after 4 days of abstinence [24]. This outcome supports the notion that shorter abstinence times may confer benefits concerning sperm motility by maintaining a higher total antioxidant capacity in seminal plasma.

Indeed, additional factors play a role in modulating sperm motility, as evidenced by a positive correlation between levels of neutral alpha-glucosidase and motility [30,31]. Elzanaty [30] reported elevated levels of neutral alpha-glucosidase in semen samples produced after 4–7 days of abstinence compared to shorter abstinence times. Although a clear benefit of short abstinence times was not consistently observed across all types of sperm pathology in our study, whenever an association was demonstrated, shorter abstinence times consistently correlated with higher motility. Therefore, shorter abstinence times can be recommended when increased motility is desired. Given that functional testing has indicated an improvement in sperm quality after a short abstinence period [24,32], further research focusing on pathologic sperm samples could provide additional insights into this matter.

### 4.3. Morphology

In our study, a shorter abstinence period was linked to a higher percentage of morphologically normal spermatozoa in both normal and OAT samples when days of abstinence were assessed as a continuous variable. Teratozoospermic samples exhibited a higher median percentage of morphologically normal spermatozoa after 0–2 days compared to 3–7 days of abstinence. Our findings align with another large cohort study, indicating that shorter abstinence periods were associated with a higher percentage of morphologically normal spermatozoa [15]. However, results varied significantly across different pathologies, with teratozoospermic samples showing optimal outcomes in the 0–2 days abstinence window, and OAT samples demonstrating a significant association with shorter abstinence time in the analysis of continuous abstinence days. The benefit of a short abstinence time was also observed in other studies investigating subnormal semen samples [6,8], but conflicting results exist, with some studies showing no association between abstinence time and changes in morphology in both normo- and oligozoospermic men [17,24,33,34]. In contrast, a study examining semen samples from men who abstained sequentially for 1–10 days reported a significant increase in normal spermatozoa after 2–5 days of abstinence and a significant decrease after 10 days [35]. As ROS levels correlate negatively with the percentage of spermatozoa with normal and borderline morphology [36], increased exposure to ROS in the epididymis might play a role in the decreasing numbers of morphologically normal spermatozoa after prolonged abstinence periods.

### 4.4. Strengths and Limitations

Our study presents data from one of the largest cohorts of men in infertile relationships. In contrast to existing research, we not only explored differences between normal and oligozoospermic semen samples but also included samples categorized as asthenozoospermic, teratozoospermic, and OAT. However, certain limitations should be considered, including the retrospective design and small cohorts in some pathological categories. Our findings may not be representative of men with cryptozoospermia or severe asthenozoospermia, as individuals with 0% motile sperm or sperm with normal morphology were excluded. Additionally, assessing semen quality through functional testing would be beneficial [37]. As most participants adhered to the advice of abstaining for 2–7 days before sample production, a few individuals had abstinence periods outside this window. Therefore, results in groups with limited sample sizes have to be carefully approached. The limited number of samples in certain categories, especially OAT samples, warrants a cautious interpretation of these results. The possibility of false responses to conform to the recommended abstinence time might lead to an underestimation of our findings. Another limitation is the cross-sectional design with only one sample per patient, neglecting potential within-subject variability. Previous studies have reported considerable variation in values for volume, concentration, motility, and percentage of normal forms between different samples from the same patient. Within-subject coefficients of variation were calculated between 25 and 28% for ejaculate volume, 26 and 29% for sperm concentration, 18 and 34% for total motility, and 19 and 29% for morphology [38,39,40]. However, the between-subject coefficients of variation exceeded the within-subject coefficients of variation in all three studies, with intraclass correlation coefficients ≥ 0.60 for most sperm parameters, indicating substantial reliability.

## 5. Conclusions

The nature and strength of associations between abstinence periods and semen quality vary based on underlying pathologies and the specific parameters investigated, making it challenging to offer clear recommendations for individual cases. If the primary goal is to improve concentration, extending the abstinence time beyond 7 days may be advantageous. However, caution is warranted to prevent excessive oxidative damage by reactive oxygen species (ROS), and we do not recommend excessively prolonged abstinence periods for this reason. For motility, a shorter abstinence time appears to be beneficial. In men diagnosed with OAT, a longer abstinence time is associated with a higher percentage of morphologically normal spermatozoa. In essence, the choice of collection strategy should align with the desired result, with longer abstinence time positively linked to concentration and shorter abstinence time associated with improved motility. Conducting more functional testing on pathologic sperm samples could further enhance our understanding and strategies for overcoming infertility.

## Figures and Tables

**Table 1:** Semen parameters.

	Normal Samples		Oligozoospermic Samples		Asthenozoospermic Samples		Teratozoospermic Samples		OAT Samples	
	N	Mean ± SD	N	Mean ± SD	N	Mean ± SD	N	Mean ± SD	N	Mean ± SD
Sperm concentration (m/mL)	1256	62.78 ± 43.37	1769	3.72 ± 4.43	1714	24.76 ± 28.33	998	24.29 ± 26.17	405	5.44 ± 4.30
Ejaculate volume (mL)	1252	3.51 ± 1.62	1748	3.42 ± 1.72	1705	3.31 ± 1.67	994	3.40 ± 1.63	404	3.51 ± 1.69
Progressively motile spermatozoa (%)	1256	49.55 ± 11.69	1164	23.72 ± 17.33	1714	17.64 ± 9.08	990	27.78 ± 17.33	405	15.10 ± 8.80
Total motile spermatozoa (%)	1256	61.92 ± 11.97	1174	35.51 ± 20.51	1714	32.69 ± 15.61	991	43.09 ± 19.50	405	28.27 ± 14.17
TMSC ^1^	1252	128.50 ± 99.70	1132	6.18 ± 8.24	1680	16.44 ± 24.85	987	41.53 ± 58.79	403	3.45 ± 4.54
Spermatozoa with normal morphology (%)	1256	14.28 ± 8.85	876	4.94 ± 5.63	1324	6.47 ± 6.82	998	1.35 ± 1.10	405	1.30 ± 1.29

^1^ TMSC = Total Motile Sperm Count.

**Table 2:** Association between abstinence time and various factors of different sperm pathologies.

	Beta	SE	*p*-Value
Independent variables			
All samples (n = 4303)			
Sperm concentration (Mio/mL)	0.149	0.001	<0.001
Progressively motile spermatozoa (%)	−0.084	0.003	<0.001
Spermatozoa with normal forms (%)	−0.033	0.005	0.030
Normal samples (n = 2381)			
Sperm concentration (Mio/mL)	0.162	0.001	<0.001
Progressively motile spermatozoa (%)	−0.057	0.004	0.005
Spermatozoa with normal forms (%)	−0.043	0.007	0.039
Oligozoospermia (n = 888)			
Sperm concentration (Mio/mL)	0.052	0.032	0.125
Progressively motile spermatozoa (%)	−0.049	0.007	0.147
Spermatozoa with normal forms (%)	−0.033	0.012	0.329
Asthenozoospermia (n = 704)			
Sperm concentration (Mio/mL)	0.130	0.003	0.001
Progressively motile spermatozoa (%)	−0.041	0.019	0.274
Spermatozoa with normal forms (%)	−0.016	0.010	0.661
Teratozoospermia (n = 1077)			
Sperm concentration (Mio/mL)	0.206	0.003	<0.001
Progressively motile spermatozoa (%)	−0.138	0.004	<0.001
Spermatozoa with normal forms (%)	0.051	0.096	0.090
Oligoasthenoteratozoospermia (n = 276)			
Sperm concentration (Mio/mL)	0.174	0.012	0.004
Progressively motile spermatozoa (%)	−0.039	0.025	0.517
Spermatozoa with normal forms (%)	0.149	0.253	0.013

Multiple regression analysis with number of days as dependent variable.

**Table 3:** Correlation between investigated semen parameters and abstinence period in normal semen samples.

Normal Samples	Abstinence Time Intervals						Overall Significance: *p*-Value	*p*-Values		
	0–2 Days		3–7 Days		>7 Days			0–2 to 3–7 Days	3–7 to >7 Days	0–2 to >7 Days
	N	Median	N	Median	N	Median				
Sperm concentration (Mio/mL)	25	50.0	1155	50.0	76	73.5	<0.001 *	1.00	<0.001 *	0.218
Volume (mL)	25	1.7	1151	3.3	76	3.7	<0.001 *	<0.001 *	0.002 *	<0.001 *
Progressively motile spermatozoa (%)	25	51.0	1155	48.0	76	46.0	0.230			
TMSC	25	56.8	1151	100.3	76	156.6	<0.001 *	<0.001 *	<0.001 *	<0.001 *
Total motile spermatozoa (%)	25	68.0	1155	62.0	76	59.0	0.087			
Spermatozoa with normal forms (%)	25	10.0	1155	12.0	76	12.0	0.926			

* Statistically significant. TMSC: Total Motile Sperm Count.

## Data Availability

Data are available upon reasonable request.
